# Integrating qualitative research methods into care improvement efforts within a learning health system: addressing antibiotic overuse

**DOI:** 10.1186/s12961-016-0122-3

**Published:** 2016-08-15

**Authors:** Corrine E. Munoz-Plaza, Carla Parry, Erin E. Hahn, Tania Tang, Huong Q. Nguyen, Michael K. Gould, Michael H. Kanter, Adam L. Sharp

**Affiliations:** 1 Department of Research & Evaluation, Kaiser Permanente Southern California, Pasadena, CA 91101 United States of America; 2 Patient Centered Outcomes Research Institute, Washington, DC, 20036 United States of America; 3 Quality and Clinical Analysis, Kaiser Permanente Southern California, Pasadena, CA 91101 United States of America

**Keywords:** Mixed methods, Qualitative research, Embedded research, Acute sinusitis, Antibiotics, Guidelines

## Abstract

**Background:**

Despite reports advocating for integration of research into healthcare delivery, scant literature exists describing how this can be accomplished. Examples highlighting application of qualitative research methods embedded into a healthcare system are particularly needed. This article describes the process and value of embedding qualitative research as the second phase of an explanatory, sequential, mixed methods study to improve antibiotic stewardship for acute sinusitis.

**Methods:**

Purposive sampling of providers for in-depth interviews improved understanding of unwarranted antibiotic prescribing and elicited stakeholder recommendations for improvement. Qualitative data collection, transcription and constant comparative analyses occurred iteratively.

**Results:**

Emerging themes and sub-themes identified primary drivers of unwarranted antibiotic prescribing patterns and recommendations for improving practice. These findings informed the design of a health system intervention to improve antibiotic stewardship for acute sinusitis. Core components of the intervention are also described.

**Conclusion:**

Qualitative research can be effectively applied in learning healthcare systems to elucidate quantitative results and inform improvement efforts.

**Electronic supplementary material:**

The online version of this article (doi:10.1186/s12961-016-0122-3) contains supplementary material, which is available to authorized users.

## Background

In two recent reports, the National Academy of Medicine defined the Learning Healthcare System as an iterative, innovative process to improve healthcare delivery and outcomes [1, 2]. Ideally, learning healthcare systems leverage advances in information technology to identify variation in services and patient outcomes, then establish a feedback loop between researchers, clinicians and leadership to rapidly evaluate and improve quality and efficiency [3–5]. Despite evidence that interventions adapted to local contexts and cultures can achieve success, there is limited practical literature describing this process [6, 7].

We report our experience within Kaiser Permanente Southern California, an integrated healthcare delivery system with 14 medical centres and over 4 million members, as an example of how qualitative and quantitative methods may be combined to support the rapid cycle improvement process [8]. This study specifically sought to understand processes and drivers associated with overuse of antibiotics for treatment of acute sinusitis, as well as to garner physician stakeholders’ recommended strategies for improving prescribing behaviours at the point of care.

Overuse of non-recommended antibiotics to treat acute sinusitis is a global healthcare problem associated with unwarranted costs and burden to health systems worldwide. For example, in the United Kingdom, more than 90% of patients presenting with acute sinusitis are prescribed antibiotics [9, 10]. Another European study of six different countries found that between 56% and 87% of acute sinusitis encounters resulted in antibiotics contrary to guidelines, thereby exposing patients to unwarranted harm and costs [11]. Reports in the United States are similar, with approximately 30 million individuals affected by acute sinusitis every year [12–14] and 85–98% receiving a prescription for antibiotics [15]. Noting limited research in the United States on the rates of inappropriate antibiotic prescribing in the outpatient setting, Fleming-Dutra et al. [16] found an estimated annual antibiotic prescription rate of 506 per 1000 population from 2010 to 2011. The authors determined that, of these, an estimated 353 prescriptions were likely appropriate and called for improvements in antibiotic stewardship. Targeting these overprescribing patterns for improvement, the American Academy of Family Practice, the American Academy of Asthma, Allergy and Immunology, the American Academy of Otolaryngology, and the American College of Emergency Physicians listed overuse of antibiotics and imaging for treatment of acute sinusitis as part of the Choosing Wisely^©^ campaign of the American Board of Internal Medicine, which is dedicated to reducing use of low-value, unnecessary healthcare practices [17].

Patient history and physical examination is key when making a diagnosis of acute sinusitis, requiring physicians to rely on their experience and judgment of presenting symptoms, rather than radiological tests, which are generally not recommended. This can result in diagnostic uncertainty and lead to potentially harmful care [11, 18]. Criteria for the diagnosis of acute sinusitis suggest that the determination should be made based on the primary symptoms of (1) purulent rhinitis and (2) facial pain [12, 19]. Physical history obtained during the patient exam should also rely on the length of symptoms, and current guidelines for the management of acute sinusitis emphasize that uncomplicated acute sinusitis lasting less than 10 days (in the absence of severe symptoms, such as facial swelling, severe fever/worsening fever) should not result in computerized tomography imaging nor an antibiotic prescription [18, 20–22]. Despite these recommendations over the last decade, there has not been much change in providers’ practice patterns and antibiotic prescribing rates [11, 14, 15].

In this manuscript, we present the qualitative results of a mixed methods study examining patterns and reasons for use of non-recommended antibiotics for treatment of acute sinusitis. Furthermore, we highlight how we used these qualitative methods to elucidate the results of a quantitative analysis using electronic medical record (EMR) data indicating widespread overuse of antibiotics for treatment. We discuss how quantitative and qualitative findings were used sequentially, and then together, to inform the design of a care improvement intervention within the context of a large healthcare system. Finally, the core components of the resulting intervention are presented. This process (using sequential mixed methodologies to inform intervention development) is an exemplar, offering a pragmatic approach for other organisations to use in understanding current best practices and potential barriers to improving healthcare within their own specific settings.

## Methods

We used an explanatory, sequential, mixed methods design in this inquiry [23]. Quantitative data were collected and analysed in phase 1, followed by qualitative data collection and analysis in phase 2. Ultimately, this rich mix of quantitative and qualitative findings from our two-phase approach informed the development of a staged, multi-faceted healthcare intervention that improved care for patients with acute sinusitis.

In the first phase of research, we conducted a retrospective, observational study of all acute sinusitis encounters (ICD-9 code 461.x) for adult health plan members [24]. Findings indicated that (1) inappropriate antibiotic prescribing was common; (2) computed tomography imaging was infrequent; and (3) emergency department encounters were less likely to result in antibiotic prescriptions compared to primary care and urgent care patient visits. Phase 1 data spurred interest in understanding the drivers of unwarranted antibiotic prescribing and processes, as well as points for intervention within the context of our large health system. The phase 1 quantitative results shaped the planned qualitative activities in phase 2 of the study in two important ways. First, because inappropriate prescribing of antibiotics was common, but computed tomography imaging use was infrequent, the focus of phase 2 shifted toward the identification of specific drivers of antibiotic prescribing patterns, as opposed to use of imaging. Second, because emergency encounters were a small proportion of encounters and less likely to result in antibiotic prescriptions, primary care and urgent care physicians were targeted for qualitative interviews.

In phase 2, the primary objective of the qualitative research was to improve our understanding of physicians’ beliefs and practices related to overuse of antibiotics for treatment of acute sinusitis, and to elicit recommendations for encouraging guideline-consistent care within the context of our large, integrated healthcare delivery system. The semi-structured interview guide (Additional file 1) examined physician stakeholders’ perspectives on the barriers and facilitators to treating patients with acute sinusitis and elicited their suggestions about how to help clinicians avoid unwarranted antibiotic prescribing and increase provider recommendation of over-the-counter symptom relief in lieu of antibiotics (as consistent with medical evidence). The qualitative research utilized a purposive sampling approach to target and recruit primary care and urgent care physicians (n = 9) for individual, face-to-face interviews [25, 26]. The Principal Investigator met with the Chiefs of Service for primary and urgent care within the health system to introduce the research and then recruited eligible providers through email. Volunteer participants met with a member of the study team trained in qualitative interviewing procedures. Each interview lasted approximately 30–45 minutes, and was audio-recorded and transcribed verbatim.

### Analytical plan for qualitative interview data

Qualitative data collection, transcription and analyses occurred iteratively. Transcription and review of early transcripts by multiple team members provided an opportunity for quality control, leading to adjustments in the interview guide questions and transcription approach. Initial coding categories were generated from the preliminary research questions and key domains of inquiry to create a ‘start list’ of structural codes [27]. The primary coder reviewed transcripts using a constant comparative method of analysis [28, 29]. During the first coding cycle, start list codes were applied to source text and then, during secondary and tertiary coding cycles of the transcripts, ‘open’ codes were applied, whereby new emergent coding categories considered salient to the goals of the research were captured. These newly emergent codes were unexpected, but grounded in the source text and, therefore, viewed as representative of important patterns and themes. Through the merging of inductive and deductive approaches, additional coding categories were generated throughout the analysis. To ensure clarity in code definitions and to improve reliability in the coding process, all codes were reviewed by study team members, revised through team consensus, and ultimately documented in a final codebook [27]. All data were managed using ATLAS.ti [30].

### Characteristics of interview participants

The characteristics of the participating physicians are summarized in Table 1. Mean age of the physicians was 45 years (SD, 8.9), and just over half were female (56%) and White (56%). Approximately three-quarters (78%) received their residency training in family medicine, with the remaining (22%) trained in internal medicine; mean years of practice experience was 15 (SD, 7.6).

**Table 1:** Primary care provider characteristics for those participating in semi-structured interviews discussing use of antibiotics for acute sinusitis (n = 9)

	Mean (SD)
Age	44.8 (8.9)
Years of experience	15.2 (7.6)
	n (%)
Sex	
Female	5 (56%)
Male	4 (44%)
Ethnicity	
Asian	2 (22%)
Black	2 (22%)
White	5 (56%)
Residency training	
Family medicine	7 (78%)
Internal medicine	2 (22%)

## Results

### Stakeholder interviews

Interviews were conducted with nine clinical stakeholders (six primary care physicians and three urgent care physicians). Data from the qualitative interviews were organised into domains, themes and sub-themes, presented in Table 2. The two domains included primary drivers of unwarranted prescribing patterns and physicians’ recommendations to improve practice patterns. The themes encompassed clinical and non-clinical factors influencing treatment decisions and patterns of care, perceptions of current guidelines, as well as multi-level (e.g. patient-, provider- and system-level) suggestions for improvement. We begin the presentation of results with illustrations of the first domain: primary drivers of unwarranted antibiotic prescribing patterns.

**Table 2:** Acute sinusitis interviews – domains, themes/sub-themes and representative quotes

DOMAIN: PRIMARY DRIVERS OF UNWARRANTED ANTIBIOTIC PRESCRIBING PATTERNS	
Themes/sub-themes	Representative quotes
Patient expectations:	
From the provider perspective, patient expectations are one of the most significant non-clinical factors driving antibiotic prescribing patterns.	*…some patients are very honest, ‘I am here for antibiotics…so those patients I wouldn’t even try to argue because they came with a made up mind and they want to go home with antibiotics… … I will spend the 10 or 15 minutes or whatever to educate and stuff, but at the end of that, if the patient is still really just persistent and unhappy and upset, I mean, you know, I have given in before.*
Patient satisfaction:	*…but just to give a medication to shut a patient up and get them out the door I think is highly inappropriate…and the problem is there are people who do that. And we have a wider responsibility than just pleasing the patient…I mean, we have doctors who get absolute fantastic, you know, ratings that their prescribing habits may be not what we would consider guideline based. …And the negative consequence is that, then, I have that patient come to me a year later and say ‘Yeah, but when I go to this doctor, I get antibiotics and then I get better.’ And so we have the double problem at that point – not only do we have the normal conversation, but we want to be respectful of our colleagues, but at the same time we have to kind of explain, ‘Well, I don’t know what was going on then, but the good news is today, it is this.’ And, but it is harder. …some patients… are like, ‘Well, I’ll just go to this doctor and they are going to write me whatever.’ And it is like, then…you can go ahead and go to that doctor because that is not me… and sometimes… our physician in charge…will actually get complaints. ‘Oh that doctor…she was mean…she wouldn’t give me the antibiotic when I asked for it.’ And it is kind of like, great, that complaint is being held against me that I actually gave appropriate medical care.*
• Patients want ‘tangible’ treatment	
Many patients reportedly want something tangible or concrete (often in their minds a ‘prescription’) when they see their doctor and physicians struggle with the desire to meet these patient expectations.	
• Member appraisal of physician/provider services satisfaction scores	
Also, while providers are split regarding how influential these scores are on antibiotic prescribing patterns, they do believe that they play a role.	
• Get antibiotics somewhere else	
Finally, providers are sometimes resigned to providing antibiotics against current guidelines because they suspect patients will seek antibiotics from another provider (and patients often threaten to do so).	
Patient/provider communication:	*…If I have known this patient for… years…I can talk her out of* [a prescription] *even if I am like supremely busy and I have many patients falling behind that is okay because I know her and she won’t mind and I know she wants the appropriate kind of treatment.*
Providers spoke at length about the challenges communicating with patients who expect antibiotics, but do not clinically need them.	
• My own patient?	
Complicating the conversation is the degree to which there is a provider and patient connection – physicians find it difficult to influence patients toward alternative treatments when they do not have an established history with the patient.	
Clinical guidelines:	*…But again, the idea that sinusitis requires decongestants, not antibiotics, is still relatively new. And usually from the time of a guideline, it takes like 10 years for implementation, but the more important thing is not the patient education, as it is the doctor education…*
While providers had a basic understanding of the guidelines and sources available, they find it challenging to stay abreast of the most current recommendations and, therefore, tend to rely more on their clinical experience for making evaluation and treatment decisions.	
	*If we read them* [the guidelines]*…if it comes to us! Because sometimes I know that there are many, many guidelines out there, it doesn’t quite come to us in a conveniently readable way, so then I wouldn’t know that it is out there.*
• Guidelines take a long time to become common, accepted practice	
• Hard to keep up with guidelines	
• Cannot find them when I need them	
DOMAIN: RECOMMENDATIONS TO IMPROVE PRACTICE PATTERNS	
Themes/sub-themes	Representative quotes
Patient level:	*We used to have a little graph for URI that says that you will not get better until about 2 weeks later – the average duration of sickness is about 17 days – so if patients come in with URI symptoms and they are asking when should I get better I will just point out this graph… And the majority of the times, if I know the patient and the patient knows me, they are pretty okay* [and say] *‘Oh really, it takes that much time for me to get better, okay, no problem, in that case you know I will just go home and chill.’*
There was consensus among providers that patients need additional education on the natural course of acute sinusitis and recommended treatment options.	
• Posters on acute sinusitis/education materials	
• Providers need ‘back-up’ from trusted sources	
Provider level:	*…but I think in terms if I am just focusing on acute sinusitis, how to manage people with acute sinusitis, two things would be helpful, physician in-service, because it is always good to learn more, and the visual card* [pocket card]*, not to put up in the exam room to aid with the patient education, more to educate ourselves, what is the appropriate thing to do…*
In addition to patient education, providers would like to see more emphasis placed on provider education, including in-service education opportunities and improved access to guidelines.	
• In-services/continuing medical education credit	
• Easier access to guidelines/recommendations	
System level:	*You know, I think, I don’t remember which situation it was, but if, perhaps with the diagnosis, you know if you had a diagnosis of sinusitis, and that was under your problem list for the day or something like that; if there was some easy way to bring up guidelines, right from there, I think that would be very useful.*
Providers generally support the idea that integrating clinical decision aids into the electronic medical record can be an effective way to impact antibiotic prescribing. Several providers explained how they use the electronic medical record system to educate patients directly for other conditions and how it could be applied in the case of acute sinusitis as well. ‘Click fatigue’ was noted as a potential barrier, but one suggestion was to allow a ‘soft stop’ in the best practice alert workflow to avoid this issue.	
	*…so similar you know, if sinusitis, if we don’t want to order antibiotics and something pops up* [in the electronic medical record] *and says you know antibiotics is not necessary in the first 14 days, do this and this at home, we can just say ‘Look, this is what is recommended’… Absolutely, it is not your personal opinion…. No, not necessarily, because you can just one click and cancel it if it is not relevant to what I am doing right now. It is always very helpful to have those, you know, not guideline, the guide – just the guide through, okay I should do this first and I should not do you know x, y, and z first. It is always helpful, because we cannot really keep up with many topics that are happening out in the world…*
• Role of electronic medical records systems	

### Primary drivers of unwarranted antibiotic prescribing patterns

Physicians underscored their desire to help patients better understand their symptoms and reasons why alternative treatments are often recommended in lieu of antibiotics for uncomplicated sinusitis. Despite this intention, physicians noted multiple barriers to following best practices within the clinical setting. These included patient expectations and perception of the relationship between prescribing patterns and patient satisfaction ratings.

#### Patients’ expectations and satisfaction

Physicians reported that patients’ expectations, and the internal and external pressures to meet those expectations, are the most important non-clinical factors driving antibiotic prescribing patterns.

Patients are described as wanting tangible action (often in their minds a ‘prescription’) when they come in for an appointment and providers report struggling with the desire to meet these expectations. The following physician shared her perspective, stating:

“*…a lot of people here are working class people, they can’t be taking off* [work] *a lot. They pay a lot of money to come in here and they feel very pissed off when they come and spend sometimes even $50 …And then they are kind of coming out like, you know, do sinus washes and stuff…So it is a real conundrum and I don’t know what to do about it.*”

Physicians pointed out that routine evaluation of their services through the Member Appraisal of Physician/Provider Services surveys distributed to patients after medical encounters are, in part, responsible for driving unwarranted antibiotic prescribing patterns as well. Speaking on this issue, one provider commented:

“…*there is one person here who is…a very big over prescriber of antibiotics, in addition to other things. Like over ordering of a lot of things.* [That physician] *probably has the best* [Member Appraisal of Physician/Provider Services] *scores in the region… Patients love* [Dr. X]*!*”

Another physician echoed the sentiment of her peers, who admitted they sometimes provide antibiotics against current guidelines because they suspect patients will seek antibiotics from another provider (and often threaten to do so):

“*A lot of times I give antibiotics against my…better judgment…either the patient has an expectation and they are very angry and I know they are just going to…see somebody else until somebody gives it to them, which is really… common.*”

These reports reveal that pressure to meet prevailing social norms and patients’ expectations (both in the office encounter and as reflected in patient satisfaction assessments) may supersede the desire to provide guideline-concordant care. This concern may be further influenced by the financial incentive providers are given to receive favourable satisfaction scores in our system.

#### Patient and provider communication

Providers spoke at length about the challenges they face communicating with patients who expect antibiotics, but do not clinically need them. This perception exists despite specific courses offered to providers designed to educate and train in effective communication skills. Limited time during patient visits to effectively convey the current recommendations is a reported challenge, complicated by the degree to which there is a provider and patient connection – physicians find it difficult to influence newer patients toward alternative treatments when they do not already have a strong, established relationship with the patient:

“*Yeah,* [patients coming in wanting antibiotics]*…that is common. I think I would say it is much, much easier to* [convince]*…my own patients, because I know them and they trust me and* [they] *know I am not withholding anything from them, you know, there is a just a better level of trust there.*

#### Access to guidelines

While physicians reported a basic understanding of the current acute sinusitis guidelines and sources available, staying abreast of the most current recommendations is perceived as challenging. In particular, physicians highlighted that integration of guidelines into practice can be slow and it can be difficult to keep up with the rapid pace at which new guidelines are published.

### Physician recommendations to improve antibiotic prescribing patterns

The second domain in this study included physicians’ recommendations to improve antibiotic prescribing patterns, which included patient-, provider- and system-level strategies.

#### Patient education

There was consensus among providers that patients need additional education on the natural course of acute sinusitis and recommended treatment options. Providers stressed the importance of being able to effectively communicate that the length of symptoms is critical and that decongestants, antihistamines and nasal washes are typically the first line of defence. In particular, providers value patient education and resource materials from trusted sources external to our health system, because they can provide ‘back up’ during discussions with antibiotic-seeking patients when watchful waiting and/or alternative treatments are the preferred course. Several providers mentioned a previous campaign in their acute care settings that placed large posters in exam rooms to educate patients about the symptomology and recommended treatment for upper respiratory infections. The following provider emphasized that similar types of educational resources could also be helpful for educating patients during acute sinusitis encounters:

“*…*[but] *my chart helps…you should make that in every office, to have a huge chart* [on the wall] *like that even bigger than the ones they make* [for upper respiratory infections]*, because that has a lot of impact on patients, and maybe some other large print things about maybe the dangers of antibiotics…It is really, it has helped me a lot in arguments with patients.*”

#### Provider education

In addition to patient education, physicians would like to see greater emphasis placed on provider education, including in-service education opportunities and improved access to guidelines. While physicians do have protected independent education time (one half day per week), several providers expressed disappointment at the loss of protected group education time within the organisation because it offered opportunities for delivering more formal curricula:

“*But there used to always be a Tuesday afternoon* [group] *education* [session]*. And I found those really valuable. You know, you get your* [Continuing Medical Education] *credit and to hear things that are updated…so we don’t have that anymore, but I found that to be the best…we get a lot of things emailed, and… we have this primary care website that I am sure has all of this stuff on there… I can’t say it is really useful.*”

#### Clinical decision aids within the EMR system

Providers generally support the idea that integrating clinical decision aids into the EMR system can be an effective way to affect antibiotic prescribing, with the caveat that a systems-level intervention of this type should also include components that target other levels of influence (e.g. patient- and provider-level components). Physicians shared numerous stories and examples of how they have used the EMR system as a direct education tool with patients, such as the primary care provider who instructed, “*I am very into the patient being a part of the decision, so I’ll turn the screen and say…these are the criteria for antibiotics…*” Thus, best-practice alerts may serve as an intervention with physicians and reinforcement for providing guideline concordant care, but also as a teaching tool for patients (e.g. by spurring physician–patient discussion and or offering quick access to patient-friendly education materials by embedding them in the best practice advisory system).

### Core components of a staged, multi-faceted healthcare intervention

Informed by the findings from phases 1 and 2 of the study, the following core intervention components were designed and implemented in order to reduce use of non-recommended antibiotics for treatment of acute sinusitis: (1) an educational presentation targeting acute care providers; (2) a system-level best practice alert within the EMR; and (3) a patient-friendly publication embedded within the best practice advisory.

#### Provider education – “Much Ado about Snot: Evaluating and Treating Acute Sinusitis”

This educational presentation targeted primary care and urgent care providers and was initially launched on two separate dates as an interactive web-based application. Prior to the launch of the web-based training for the broader Kaiser Permanente Southern California audience of providers, the content was presented to the clinical leaders in our health system. Based on our qualitative findings that physicians find it challenging to stay abreast of current recommendations and desire better integration of guidelines into practice, learning objectives of the course were to (1) understand current recommendations for diagnosing acute sinusitis; (2) identify how best to treat acute sinusitis; and (3) incorporate best evidence into current practice. After the original web-based presentation sessions were delivered, the training was uploaded onto the physicians’ online education portal, where it remains available to providers.

#### System-level EMR best practice alert (BPA)

The BPA is triggered within the EMR for patients over 18 years of age with an encounter diagnosis of acute sinusitis, when antibiotics are prescribed during the clinical encounter. The EMR-based electronic alert acts as a reminder to clinicians about guideline recommendations for treatment of acute sinusitis, and requests information about the requested antibiotic prescription. The system alert also guides providers to quickly select the recommended antibiotic when appropriate. The alert audience includes all providers with prescribing authority in the outpatient setting. Rollout of the BPA was staggered across medical centres over several months to facilitate comparisons between pre- and post-implementation groups.

#### BPA-embedded patient education materials

The BPA was designed based on information gathered during the qualitative interviews. Providers requested support from reputable, nationally recognized, third-party entities to help with patient discussions with those seeking unwarranted antibiotics. The research team identified the Choosing Wisely® publication entitled, *Treating Sinusitis: Don’t Rush to Antibiotics*, as an effective patient education resource made available during the clinical encounter in the BPA. This Choosing Wisely^®^ publication, which is a joint publication with Consumer Reports^®^ and the American Academy of Family Physicians, emphasizes current recommendations and outlines a number of over-the-counter and home remedy options as alternatives to taking antibiotics for a likely viral infection. This publication remains embedded within the BPA, such that providers can readily access the handout and provide it to patients during an acute sinusitis encounter.

### Intervention implementation

The design of the intervention was based on our qualitative results, which directly informed our efforts to successfully reduce use of non-recommended services at the system-level (BPA embedded in the EMR), provider-level (physician education presentation), and patient-level (BPA-embedded patient education materials). Results from our evaluation of this intervention are forthcoming, and preliminary findings are encouraging.

## Discussion

Qualitative interviews with clinicians elicited valuable information and expanded our understanding of the practice context in ways that we could not have achieved exploring the structured EMR data alone. Retrospective, structured data collected during phase 1 of our research suggested an opportunity to develop targeted implementation strategies to improve antibiotic stewardship and to translate accepted antibiotic guidelines across care settings. Whereas the first phase of the research indicated a clear gap between current practice and best practices for acute sinusitis, the qualitative component of our mixed methods approach explored why the gap exists, and suggested how to bridge the gap by examining the barriers and/or facilitators to implementing these best practices in our specific care settings.

Interviews with primary and urgent care providers underscored the extent to which physicians’ concerns about patients’ expectations and satisfaction drive unwarranted antibiotic prescribing practices. Physicians emphasized the difficulty conveying the option and potential efficacy of evidence-based, medically indicated treatment alternatives (e.g. watch and wait, over-the-counter medications) to patients intent on receiving antibiotics, but for whom a prescription is not appropriate. Patient co-pays (typically ranging from $20 to $50 per visit) and the linking of patient satisfaction scores with provider incentives may further complicate communication, arguably redefining the patient–provider relationship as a customer–provider relationship. According to provider interviews, these factors systematically reinforce undesired behaviour, with physicians openly admitting that pressure from patients and incentive structures have caused them to prescribe antibiotics contrary to known guidelines.

Physicians articulated a need for easily-accessible guidelines, patient education resources, and enhancements to the EMR system as important tools to reinforce their conversations with patients. Recent literature in primary care settings suggests that provider concerns regarding the negative influence of patient satisfaction on unwarranted antibiotic prescribing may be real. In a cross-sectional analysis of national patient survey data and prescribing data within primary care practices in England, patients reported higher rates of dissatisfaction with their care if they were associated with a practice less likely to prescribe antibiotics [10]. Researchers suggest that acknowledging the extent to which trade-offs may exist between the promotion of physician–patient relationships and antibiotic prescribing is an important next step, and call for additional studies that can determine ways that patient satisfaction can be maintained during visits where providers refuse to prescribe antibiotics based on sound clinical evidence [10, 31].

### Limitations

The fundamental goals for conducting interviews was to help us to (1) explain the quantitative data by providing rich context to the patterns identified; (2) focus on the departments/physicians in our setting most likely to overprescribe unwarranted antibiotics; and (3) uncover any potential leverage points for intervention. The nine interviews were detailed, generating 157 pages of written transcript containing in-depth, deeply contextualized data from providers within our health system. Spending a significant portion of our interview time with physicians discussing both the clinical and non-clinical factors that impact their treatment decisions, we found a high degree of consensus among physicians as to the primary drivers of unwarranted antibiotic prescribing behaviours. After the first two to three initial interviews, repetition of thematic classifications was evident, allowing the team to group participants’ responses into key emerging thematic categories contextualized to our specific healthcare setting, including (1) the role of patient expectations/satisfaction; (2) the value of the patient–provider relationship to antibiotic prescribing, yet the barriers to achieving effective patient–provider communication within our setting; and (3) the need for improved access to updated guidelines. Providers’ suggestions for how to address the problem also converged quickly, emphasizing the key thematic groupings as (1) a desire for patient education materials from trusted external sources to serve as ‘back up’ with patients, (2) provider reinforcement through in-service education, and (3) capitalizing on the EMR as a tool for both provider reinforcement and patient education. These themes were consistent throughout the remaining interviews, with few divergent viewpoints emerging.

However, we acknowledge that the qualitative findings reported herein may not be generalizable to all healthcare delivery systems. Physician participants were recruited to the phase 2 portion of the study using purposive, convenience sampling and the qualitative sample of interviews was relatively small. Despite these limitations, acute care providers in our integrated health system, which serves over 4 million members, maintain patient panels that are socio-demographically diverse and largely representative of the general population of Southern California [32]. While it is possible that the physicians’ views expressed during the interviews may not be representative of the perceptions of all providers within our system, we remain less concerned that they represent widely different patient populations from physicians who did not participate in the qualitative component. In addition, we achieved reasonable variation in our provider sample, in terms of their characteristics across gender, age, race/ethnicity, and years of experience.

Given our team’s embedded nature within the organisation, it is also possible that the physicians we interviewed gave socially desirable responses out of self-awareness or an attempt to please the interviewer. However, two observed factors are likely to mitigate these concerns. First, the major qualitative themes, namely that physicians report difficulty communicating with antibiotic-seeking patients about evidence-based alternatives and that physicians face pressure to meet patient expectations, are consistent with the findings from our quantitative data, which revealed high rates of antibiotic prescribing, and only about one-third of patients receiving recommended care for acute sinusitis. Second, as with any qualitative research, the overall aim in terms of sample size is to reach saturation; namely, the point at which no new information is emerging from the data [33]. As we noted above, despite our relatively small sample size for interviews, the suggested drivers that physicians ascribed to inappropriate antibiotic prescribing patterns, and their subsequent recommendations for potential intervention, became quickly saturated during the interview process.

Further, it was not feasible in this small, exploratory study to interview patients in addition to physicians (both those who did or did not receive antibiotics during an acute sinusitis encounter). Certainly, speaking with patients would provide an opportunity to examine their opinions and the extent to which they agree or disagree with physicians’ perceptions of their needs and satisfaction as drivers of unnecessary antibiotic prescribing. Realistically, however, we believe that many health systems similar to ours will also lack resources to conduct large numbers of interviews due to funding and time constraints; this is another reason that our study is likely to be a useful, pragmatic example worthy of sharing outside of our health setting.

Finally, a pragmatic consideration when embedding qualitative research into a learning health system is to recognize and address when provider perceptions are at odds with evidence. For example, we found that some providers support integrated decision support, but experience and evidence suggests that these interventions are commonly disregarded and may offer limited benefits [34, 35]. Within learning healthcare organisations, the perception of issues raised through qualitative investigation must be balanced with sound evidence and previous experience. Regardless, even when stakeholders’ perceptions may be false, identifying, understanding and addressing them through informed design and implementation may facilitate future success.

## Conclusion

Our work offers an example of the benefits of embedded research, defined as work using operational funds to integrate scientific methods into projects addressing the practical needs of an organisation to provide generalizable knowledge. Therefore, although antibiotic stewardship and mixed methods studies are not alone novel, this paper presents a new model of research that incorporates providers’ viewpoints and contributes to the interpretation and validation of quantitative findings. We found that embedded qualitative research not only improved our understanding of quantitative findings, but efficiently moulded our intervention in ways that would not have been possible otherwise. Phase 2 of our research contributed to our ability “*…to understand and work in ‘real world’ or usual practice settings, paying particular attention to the audience that will use the research, the context in which implementation occurs, and the factors that influence implementation*” [36, Summary Points figure, point 3].

We postulate that qualitative research can provide a clearer understanding of factors influencing practice patterns at the point of care and lead to more effective healthcare delivery interventions [7, 37–41]. Furthermore, as ubiquitous use of EMR systems offers improved opportunities for quantitative analytics, embedding complementary qualitative methods into improvement efforts is possible, necessary, and likely to lead to more efficient interventions and better care delivery. Collaboration with clinical and operations leaders throughout this process offered rapid ability to apply findings within improvement efforts. Ultimately, our experience leveraging qualitative methods to target low-value antibiotic use can be a model for other learning healthcare systems undertaking care improvement efforts, since these methods can be readily applied to other diseases, specialties and stakeholders to successfully broaden the evaluation of clinical challenges at the point of care.

## Additional file

Additional file 1: Semi-structured interview guide. (DOC 32 kb)
