# Supplementary material for: Integrating qualitative research methods into care improvement efforts within a learning health system: addressing antibiotic overuse
Source: Health Res Policy Syst. 2016 Aug 15;14:63. doi: 10.1186/s12961-016-0122-3 (PMC4986245; doi:10.1186/s12961-016-0122-3)
Supplement: Additional file 1: — Semi-structured interview guide. (DOC 32 kb) [file 12961_2016_122_MOESM1_ESM.doc]

**Appendix: Semi-structured Interview Guide**

**ACUTE SINUSITIS INTERVIEW GUIDE**

**Case Study Vignette**

To begin, we would appreciate it if you would take a few minutes to review the following patient vignette. Once you are finished reading about the patient’s case, I will ask you several related follow-up questions.

[Interviewer instructions: provide the participant with a printed copy of the case study].

**Patient: “Donna,” 43 year old female.**

**History/Presenting Illness.** Patient presents today with 5 days of nasal congestion. She has borderline Type 2 diabetes, but no other chronic medical conditions. Associated symptoms include cough, fever/malaise and face pain. Pertinent negatives include no chest pain, abdominal pain, nausea/vomiting, dysuria, ear pain, headaches, or swollen glands. Patient reports that this is a new problem and that she has tried nothing for the symptoms.

**Physical Exam**. Constitutionally, she is oriented to person, place, and time. She appears well-developed and shows no signs of distress. Physical exam findings include right and left maxillary sinus tenderness.

**Case study follow-up questions:**

*C1. How would you typically approach evaluating and treating a patient like Donna? Do you think most of your physician colleagues would approach this case similarly? Why or why not?*

*C2. What specific history or exam finding(s), if any, would change your approach to evaluation and treatment in this case?*

**Interview Questions**

**Section I: Clinical Factors Influencing Evaluation and Treatment Decisions**

We would like to begin the interview by asking you to reflect on clinical factors that influence how you identify, diagnose and treat patients for acute sinusitis.

Q1.1 Please describe how you initially identify when a patient has AS.

Q1.2 In your experience, what are the most important factors physicians consider when diagnosing AS?

Probe(s):

- What is it specifically that makes these factors the most important for diagnosing AS?
- Of these factors, do you rely on one more than the others for determining if a patient has AS?
- How does the length of symptoms play a role in the management of AS?
- What particular challenges do providers face when trying to identify if a patient has AS? If challenges exist, please explain in detail. How do you overcome these challenges? Strategies?

Q1.3 What criteria do you use for determining which of your patients diagnosed for AS need antibiotics?

Probe(s):

- How do you determine which specific antibiotic to prescribe to your AS patients?

Q1.4 What criteria do you use to determine which of your AS patients require a CT scan?

Q1.5 Thinking about your AS patients, which individuals are most likely to benefit from a referral to a specialist?

Probe(s):

- What is it about these patients that make them most likely to benefit from seeing a specialist?
- For example, can you think of a particular AS patient (no names, please) that you recently treated and referred to a specialist? If so, can you tell me more about that patient?

**Section II: Non-Clinical Factors Influencing Evaluation and Treatment Decisions:**

Great, I would now like to explore what, if any, non-clinical factors influence your patient care decisions related to acute sinusitis.

Q2.1 Which non-clinical factors impact your treatment decisions for acute sinusitis?

Probe(s):

- - Systemic (lack of time?)
  - Vague care guidelines
  - Poor decision aids
  - Patient satisfaction
  - Difficult patient history or presentation

Q2.2 High practice volume has been shown to increase the likelihood of providers prescribing unnecessary antibiotics. How does practice volume impact your antibiotic prescribing?

Q2.3 How do patient expectations impact your care decisions for acute sinusitis?

Probe(s):

- Are there specific expectations that patients have when it comes to AS treatment? If so, what are some typical patient expectations?
- How do you deal with patient expectations that aren’t in line with your care decisions? Are there specific strategies you employ?

**Section III: Clinician Perceptions of Current Practice Guidelines**

Next, I would like to get your perspective on current practice guidelines for patients that present with acute sinusitis.

Q3.1 How helpful are the current practice guidelines when diagnosing, evaluating, and treating patients for AS in your own practice?

Q3.2 What, in your opinion, are the strengths of current practice guidelines?

Probe(s):

- Of the strengths you have mentioned, what do you think is the biggest strength of the current guidelines?

Q3.3 What, if any, limitations are there to the current practice guidelines?

Probe(s):

- Of the limitations you have mentioned, what do you think is the biggest limitation?

Q3.4 To what extent do you share AS guideline information with patients?

Probe(s):

- How often do patients proactively ask you about guidelines for AS treatment?
- If you don’t often discuss guidelines with patients, what is the primary reason? Lack of time? Patient disinterest? Other?

**Section IV: Recommendations**

We are coming to the end of the interview, but I want to take a few more minutes to give you an opportunity to offer any recommendations and/or suggestions you have regarding acute sinusitis evaluation and treatment.

Q4.1 What could be done to help providers evaluate and treat patients with AS?

Q4.2 To what extent do you think that the evaluation and treatment of AS should be a priority for KP? Please tell me why you think that.

Q4.3 Please share any ideas about how you think that Health Connect (i.e., electronic prompts) can be used as a tool for assisting providers?

**END THE INTERVIEW**: That concludes all of my questions for today. Do you have anything else that you would like to add to the discussion before we wrap up? Again, I want to thank you very much for your participation in this interview.
